# Clinical Benefit of Autologous Stem Cell Transplantation for Patients with Multiple Myeloma Achieving Undetectable Minimal Residual Disease after Induction Treatment

**DOI:** 10.1158/2767-9764.CRC-23-0185

**Published:** 2023-09-06

**Authors:** Jiahui Liu, Wenqiang Yan, Huishou Fan, Jingyu Xu, Lingna Li, Chenxing Du, Xuehan Mao, Yuting Yan, Yan Xu, Weiwei Sui, Shuhui Deng, Shuhua Yi, Kenneth C. Anderson, Lugui Qiu, Dehui Zou, Gang An

**Affiliations:** 1 State Key Laboratory of Experimental Hematology, National Clinical Research Center for Blood Diseases, Haihe Laboratory of Cell Ecosystem, Institute of Hematology & Blood Diseases Hospital, Chinese Academy of Medical Sciences & Peking Union Medical College, Tianjin, P.R. China.; 2 Tianjin Institutes of Health Science, Tianjin, P.R. China.; 3 Fujian Institute of Hematology, Fujian Provincial Key Laboratory on Hematology, Fujian Medical University Union Hospital, Fuzhou, P.R. China.; 4 LeBow Institute for Myeloma Therapeutics and Jerome Lipper Center for Multiple Myeloma Center, Dana-Farber Cancer Institute, Harvard Medical School, Boston, Massachusetts.

## Abstract

Attaining undetectable minimal residual disease (MRD) is the current therapeutic goal for multiple myeloma. But there is a current lack of data regarding the clinical benefit of autologous stem cell transplantation (ASCT) for patients with myeloma achieving early MRD-negative status after induction treatment, in addition to the interaction of longitudinal MRD status with ASCT. The current study included 407 patients with transplant-eligible multiple myeloma with available MRD status from the National Longitudinal Cohort of Hematological Diseases in China (NCT04645199), of whom 147 (34.4%) achieved early undetectable MRD and 182 (44.7%) received ASCT. Early MRD-negative status was associated with a lower risk of disease progression [HR = 0.447; 95% confidence interval (CI), 0.333–0.600; *P* < 0.001] and death (HR = 0.473; 95% CI, 0.320–0.700; *P* < 0.001). Of note, patients who achieved undetectable MRD early still benefitted from ASCT, with a remarkable improvement in the median MRD-negative duration (33.5–58.0 months, *P* < 0.001), progression-free survival (PFS; 46.0–88.3 months, *P* < 0.001), and overall survival (OS; 76.4 months to not reached, *P* = 0.003). These clinical benefits were more pronounced in patients with aggressive features (high-risk cytogenetic abnormalities or high tumor burden) compared with standard-risk patients. Similar results were observed in patients with detectable MRD after induction treatment. In addition, we identified four MRD-status transformation patterns following ASCT, which were strongly correlated with diverse survival outcomes (*P* < 0.001). Our study revealed the enhanced clinical significance of ASCT in patients with transplant-eligible myeloma, regardless of early MRD status, particularly for high-risk patients. Subsequent prospective trials are essential to validate these observations.

**Significance:**

Achieving and maintaining undetectable MRD is the current treatment goal for multiple myeloma. Our results emphasized the remarkable clinical benefit of ASCT on MRD-negative duration, PFS, and OS in patients with multiple myeloma regardless of early MRD status. These favorable impacts were more evident in patients with aggressive features. Importantly, dynamic MRD monitoring among ASCT could facilitate personalized stratification of therapeutic approaches.

## Introduction

Minimal residual disease (MRD) refers to a low level of residual malignant tumor cells that remain after treatment in patients with multiple myeloma and is considered the root cause of recurrence and relapse (1–3). As a result, attaining and sustaining MRD-negative status is crucial in achieving functional cure in multiple myeloma (4). Historically, autologous stem cell transplant (ASCT) is considered the treatment cornerstone for total therapy in multiple myeloma and has been reported to effectively enhance remission depth and duration (5–9). Nevertheless, an increasing number of patients with myeloma could achieve MRD-negative status early before ASCT due to the advanced application and combination of multiple novel agents utilized as induction treatment (10, 11). Therefore, it is unclear whether ASCT could improve the prognosis of patients with multiple myeloma without detectable MRD after induction treatment, resembling “potential curative status.”

Recently, multiple studies have assessed the prognostic significance of MRD status at 3 months after ASCT (1, 3, 12, 13) and other timepoints during maintenance therapy (12, 14–16). These studies confirmed that undetectable MRD is a powerful predictor of good prognosis and gave an understanding of the importance of the temporal profile of MRD status on prognosis. However, to date, there are a limited number of studies investigating the relationship between early MRD status and ASCT, and there are no data describing MRD conversion patterns prior to and after ASCT.

National Longitudinal Cohort of Hematological Diseases in China (NCT04645199) is one of the largest retrospective studies conducted to evaluate real-world therapeutic options and prognoses in patients with multiple myeloma. On the basis of the study design, we aimed to evaluate the clinical role of ASCT in patients with distinct MRD statuses after induction of treatment, its interaction with aggressive features, and the clinical significance of longitudinal MRD status conversion pre- and post-ASCT.

## Materials and Methods

### Patients

This study is based upon data from the National Longitudinal Cohort of Hematological Diseases in China (NCT04645199); in brief, it is a large-scale prospective cohort study that set out to analyze the incidence and risk factors associated with hematologic diseases, in addition to the treatment options and prognosis for these patients in China. This study was approved by the Blood Diseases Hospital in compliance with the Declaration of Helsinki. Written informed consent was obtained from all patients. According to the exclusion criteria, transplant-ineligible patients, non–first-line ASCT transplant-eligible patients, and those without MRD data after induction of treatment were excluded. As such, a total of 407 patients with transplant-eligible newly diagnosed multiple myeloma (NDMM) diagnosed between January 2013 and December 2019 were included in the study (Supplementary Fig. S1). All enrolled patients received four to six courses of induction treatment with novel therapeutic agents [proteasome inhibitors (PI/(immunomodulators) IMiD], following by either sequential ASCT or two to four courses of combination consolidation treatment based on their individual preferences and decisions. Patients finally underwent lenalidomide/thalidomide for at least 2 years as maintenance treatment. The diagnostic and response criteria of multiple myeloma complied with the International Myeloma Working Group (17).

### MRD and Molecular Risk Assessment

Bone marrow aspirates were obtained at various timepoints, including for example once every two courses of treatment during induction treatment, once every 3 months after ASCT/consolidation treatment, and when disease progression occurred. MRD was detected by multiparameter flow cytometry (January 2013 to December 2017) or the EuroFlow method (January 2018 to now). MRD-negative status was confirmed if at least two consecutive bone marrow flow cytometry tests detected 2 × 10^5^ events and phenotypic abnormalities in clonal plasma cells consisted of less than 20 events (0.01%, 10^−4^), with a median MRD detection sensitivity of 4 × 10^−5^ (range: 1.3 × 10^−6^ to 1 × 10^−4^). The MRD-negative duration was defined as the duration from an MRD-negative test to the first MRD-positive test or whenever the MRD test was last negative.

Bone marrow puncture was performed on patients with NDMM at diagnosis. Mononuclear cells were isolated before plasma cells were sorted with CD138 magnetic beads, and FISH detection was performed. Abnormal cytogenetic detection included 1q21 gain, 13q deletion, 17p deletion, and IgH translocation: t(11; 14), t(4; 14), t(14; 16), and t(14; 20). The positive threshold of the translocation probe was defined as 10%, and the copy-number probe was 20%. High-risk cytogenetic abnormalities (HRCA) included deletion of 17p, gain of 1q, t(4; 14), t(14; 16), and t(14; 20). The standard risk (SR) group was defined as patients without any HRCA, high-risk patients had one HRCA, and ultra-high-risk (UHR) patients had more than one HRCA.

### Statistical Analysis

Progression-free survival (PFS) was defined as the time from disease diagnosis to disease progression, death, or last follow-up. Overall survival (OS) was defined as the time from disease diagnosis to death or last follow-up. Statistical analyses were performed using the SPSS software (version 26.0; IBM) or R version 4.1.2. The survival discrepancy of diverse groups was described using the Kaplan–Meier method. The Cox proportional hazards model was utilized to compare patients in different groups and to estimate HRs and 95% confidence intervals (CI). Subgroup analyses were performed to estimate the prognostic role of ASCT. Univariate and multivariate analyses were used for PFS, OS, and MRD negativity duration, considering International Staging System (ISS) stage, lactate dehydrogenase (LDH) level, cytogenetic abnormalities, induction treatment, MRD status, and ASCT. Continuous variables for baseline features were expressed as median (range), and the rank-sum test was used for comparisons. Categorical variables were described as proportions (percentages), and the *χ*^2^ test or Fisher exact probability methods were used for comparisons between groups. A *P* value <0.05 was considered statistically significant in all analyses.

### Data Availability Statement

The data generated in this study are available upon request from the corresponding author.

## Results

### Patient Features and Treatments

In the current study, a total of 407 transplant-eligible patients with an informative MRD status were included. The median age was 55 years, 226 patients (55.5%) were male. Moreover, 48.6% and 26.5% of patients were classified as ISS III stage and the Revised ISS stage (R-ISS) III stage, respectively. With a median follow-up time of 40.6 months (range: 5.6–113.6 months), the median OS in this cohort was 78.8 (95% CI, 66.5–91.1) months.

Referring to the MRD status after induction treatment, 147 patients achieved early MRD-negative status. Of these, 72 patients subsequently received ASCT and the other 75 patients received consolidation treatment according to the original protocol. Moreover, the remaining 260 patients were MRD-positive after induction treatment, among these, 110 cases received ASCT and the other 150 cases were treated with the original consolidation therapy regimen (Supplementary Fig. S1). Next, early MRD-positive (*n* = 260) or MRD-negative (*n* = 147) groups were categorized by MRD status after induction treatment for comparative analysis. A comparison between the two groups revealed no significant difference in most baseline characteristics and treatments (Table 1). Interestingly, early MRD-negative patients had a higher proportion of R-ISS III stage associated with a 17p deletion compared with the MRD-positive cohort (*P* < 0.05). Furthermore, there were no significant differences in baseline features and MRD detection methods between patients who underwent ASCT and those who did not (Supplementary Table S1).

**TABLE 1:** Patient and treatment characteristics by MRD status after induction treatment

Characteristics (%)	All cohort (*n* = 407)	MRD-negative (*n* = 147)	MRD-positive (*n* = 260)	*P*
Age (median; years)	55 (22–65)	54 (31–65)	55 (22–65)	0.514
Sex	M: 226 (55.5), F: 181 (44.5)	M: 79 (53.7), F: 68 (46.3)	M: 147 (56.5), F: 113 (43.5)	0.585
M-component IgG IgA Light chain Others	194 (47.7) 87 (21.4) 88 (21.6) 38 (9.3)	74 (50.3) 34 (20.1) 26 (17.7) 13 (8.9)	120 (46.2) 53 (20.4) 62 (23.8) 25 (9.6)	0.497
ISS staging I II III Missing	74 (18.3) 134 (33.1) 197 (48.6) 2	19 (13.1) 52 (35.9) 74 (51.0) 2	55 (21.2) 82 (31.5) 123 (47.3) 0	0.129
R-ISS staging I II III Missing	53 (13.1) 244 (60.4) 107 (26.5) 3	13 (9.0) 85 (58.6) 47 (32.4) 2	40 (15.4) 159 (61.4) 60 (23.2) 1	0.048
Cytogenetic abnormality Del(17p) t(4;14) t(14;16) Gain 1q	56/402 (13.9) 77/387 (19.9) 17/386 (4.4) 215/405 (53.1)	28/145 (19.3) 33/143 (23.1) 7/142 (4.9) 78/147 (53.1)	28/257 (10.9) 44/244 (18.0) 10/244 (4.1) 137/258 (53.1)	0.024 0.238 0.798 1.000
HRCAs 0 1 ≥2 Missing	134 (34.6) 160 (41.4) 93 (24.0) 20	45 (31.5) 57 (39.9) 41 (28.7) 4	89 (36.5) 103 (42.2) 52 (21.3) 16	0.246
Induction treatment PIs based IMiDs based PIs+IMiDs based	299 (73.5) 7 (1.7) 101 (24.8)	111 (75.5) 3 (2.0) 33 (22.5)	188 (72.3) 4 (1.5) 68 (26.2)	0.674
ASCT	182 (44.7)	72 (49.0)	110 (42.3)	0.213

Abbreviations: ASCT, autologous stem cell transplant; F, female; HRCAs: high risk cytogenetic abnormalities, including Del (17p), t(4;14), t(14;16), or Gain 1q; IMiDs: immunomodulators; ISS: International Staging System; M, male; PIs: proteasome inhibitors; R-ISS: Revised International Staging System.

### Impact of ASCT on Survival Outcomes by Early MRD Status

**FIGURE 1:**
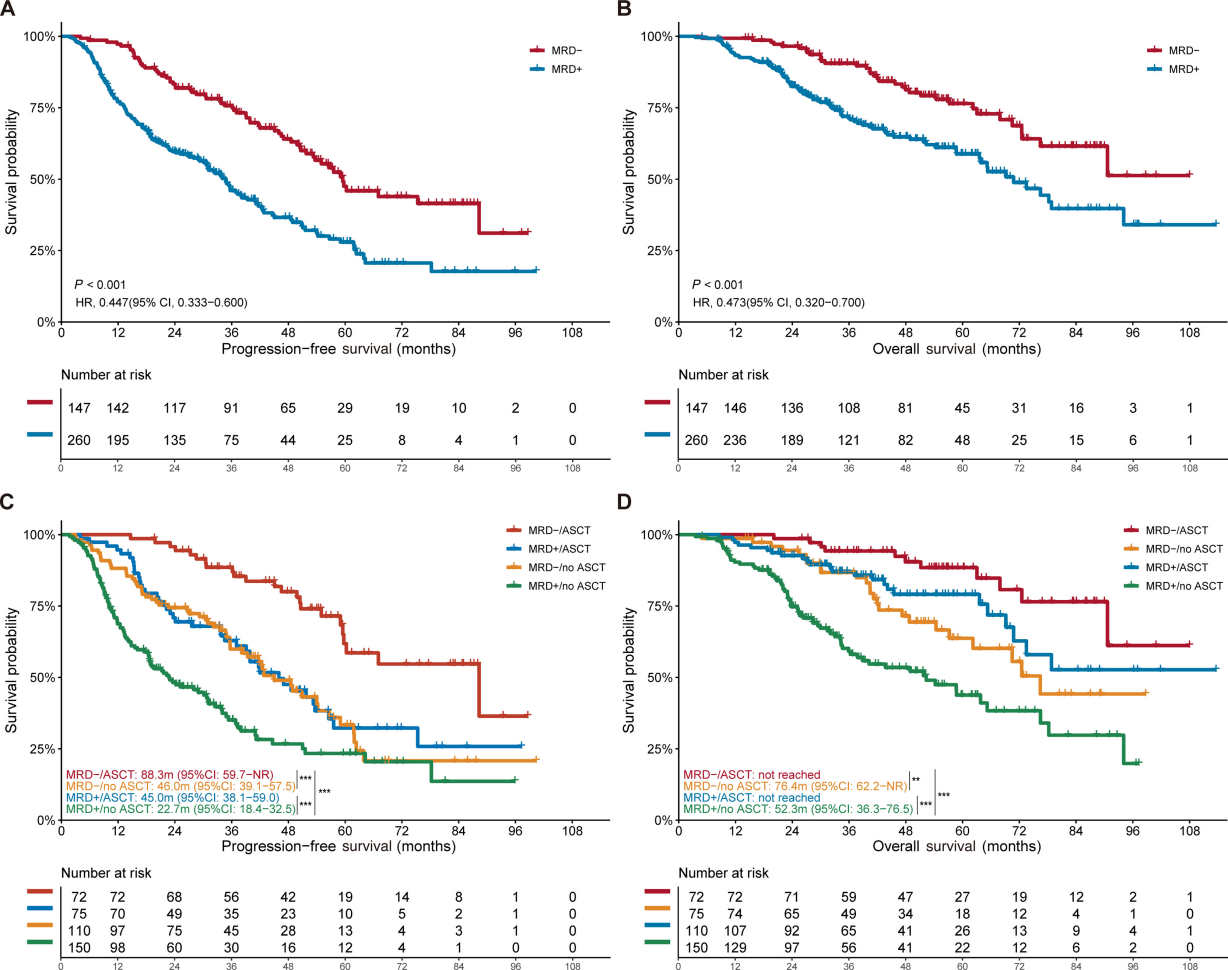
The impact of ASCT and early MRD status on prognosis. Impact of early MRD-negative status on PFS (**A**) and impact of early MRD-negative status on OS (**B**); impact of ASCT by different MRD status on PFS (**C**); impact of ASCT by different MRD status on OS (**D**). ASCT: autologous stem-cell transplant; early MRD status: MRD status after induction treatment; NR: not reached; NS: not significant; OS: overall survival; PFS: progression-free survival; *, *P* < 0.05; **, *P* < 0.01; ***, *P* < 0.001.

Patients who achieve MRD-negative status after induction treatment were at a lower risk of disease progression or relapse (HR = 0.447; 95% CI, 0.333–0.600; *P* < 0.001) and had significantly longer median PFS than the MRD-positive population (59.5 vs. 34.7 months; *P* < 0.001; Fig. 1A). The median OS was not achieved in the MRD-negative group, and a median OS of 70.8 months was observed in the MRD-positive group (HR: 0.473; 95% CI, 0.320–0.700; *P* < 0.001; Fig. 1B). Similarly, the patients who received ASCT also exhibited a considerable improvement in PFS and OS compared with those in non-ASCT group (*P* < 0.001; Supplementary Fig. S2).

Regardless of MRD status after induction treatment, ASCT was strongly associated with a longer PFS in comparison with those individuals not treated with ASCT (Supplementary Table S2). For those patients who were MRD-negative before transplant, ASCT was associated with a remarkable improvement in median PFS, from 46.0 months without ASCT to 88.3 months with ASCT (HR: 0.373; 95% CI, 0.223–0.623; *P* < 0.001; Fig. 1C). Likewise, ASCT was strongly associated with the advancement of the median OS from 76.4 months without ASCT to median OS not reached with ASCT (HR: 0.337; 95% CI, 0.164–0.692; *P* = 0.003; Fig. 1D). In those patients with detectable MRD after induction treatment, ASCT was similarly linked with an improvement in PFS (45.0 vs. 22.7 months; HR: 0.550; 95% CI, 0.396–0.763; *P* < 0.001; Fig. 1C) compared with those patients who did not receive ASCT. Moreover, the impact of ASCT on the median OS was consistent (not reached vs. 52.3 months; HR: 0.356; 95% CI, 0.223–0.568; *P* < 0.001; Fig. 1D). In addition, ASCT could overcome the adverse effect of early MRD-positive status in some patients (MRD-negative/no ASCT vs. MRD-positive/ASCT: PFS, *P* = 0.78; OS, *P* = 0.28). Of note, the prognoses of patients with early MRD-negative status + ASCT were far superior to the other three groups (*P* < 0.001). A Cox regression multivariable analysis of PFS and OS, baseline LDH level, cytogenetic abnormalities at diagnosis, early MRD status, and ASCT revealed an independent favorable role (Table 2; Supplementary Table S3). Overall, these results highlight the crucial role of ASCT in multiple myeloma regardless of MRD status after induction treatment.

**TABLE 2:** Multivariate analyses of PFS and OS in 407 patients with transplant-eligible myeloma

	PFS		OS	
Factors	HR (95% CI)	*P*	HR (95% CI)	*P*
ISS stage at diagnosis II vs. I III vs. I	1.035 (0.666–1.610) 1.339 (0.882–2.032)	0.878 0.170	0.753 (0.401–1.414) 1.645 (0.932–2.903)	0.377 0.086
Serum LDH level at diagnosis Abnormal vs. normal	1.859 (1.355–2.550)	<0.001	2.075 (1.378–3.126)	<0.001
Cytogenetic abnormality at diagnosis One HRCAs vs. SR ≥2 HRCAs vs. SR	1.356 (0.965–1.906) 1.625 (1.109–2.382)	0.080 0.013	1.431 (0.899–2.276) 2.408 (1.461–3.968)	0.130 <0.001
MRD status after induction treatment Negative vs. positive	0.450 (0.332–0.610)	<0.001	0.424 (0.282–0.637)	<0.001
ASCT status Yes vs. no	0.464 (0.347–0.621)	<0.001	0.309 (0.205–0.467)	<0.001

Abbreviations: Abnormal LDH, lactate dehydrogenase>247 U/L; ASCT, autologous stem cell transplantation; HR, hazard ratio; HRCAs: high-risk cytogenetic abnormalities, including Del(17p), t(4;14), t(14;16), or Gain 1q; ISS, International staging system; SR, standard risk, without any HRCAs.

### Impact of ASCT and Early MRD Status in Distinct Molecular Risk Cohorts

Given the adverse effects of cytogenetic abnormalities, we further evaluated the impact of early MRD status and ASCT in different molecular risk groups. For aggressive patients classified as high-risk/ultra-high-risk, the remarkably improved trend of survival outcomes for patients in MRD-negative + ASCT cohort were very similar compared with those in the whole cohort (*P* < 0.001; Supplementary Fig. S3A–S3D). Despite MRD status after induction treatment, ASCT could retain its favorable prognostic effect in patients with HRCAs (*P* < 0.05). However, in the SR cohort, the associations between MRD status and ASCT with prognosis were not as strong. The patients without HRCAs did not further benefit from ASCT regardless of early MRD status after induction treatment (Supplementary Fig. S3E and S3F).

**FIGURE 2:**
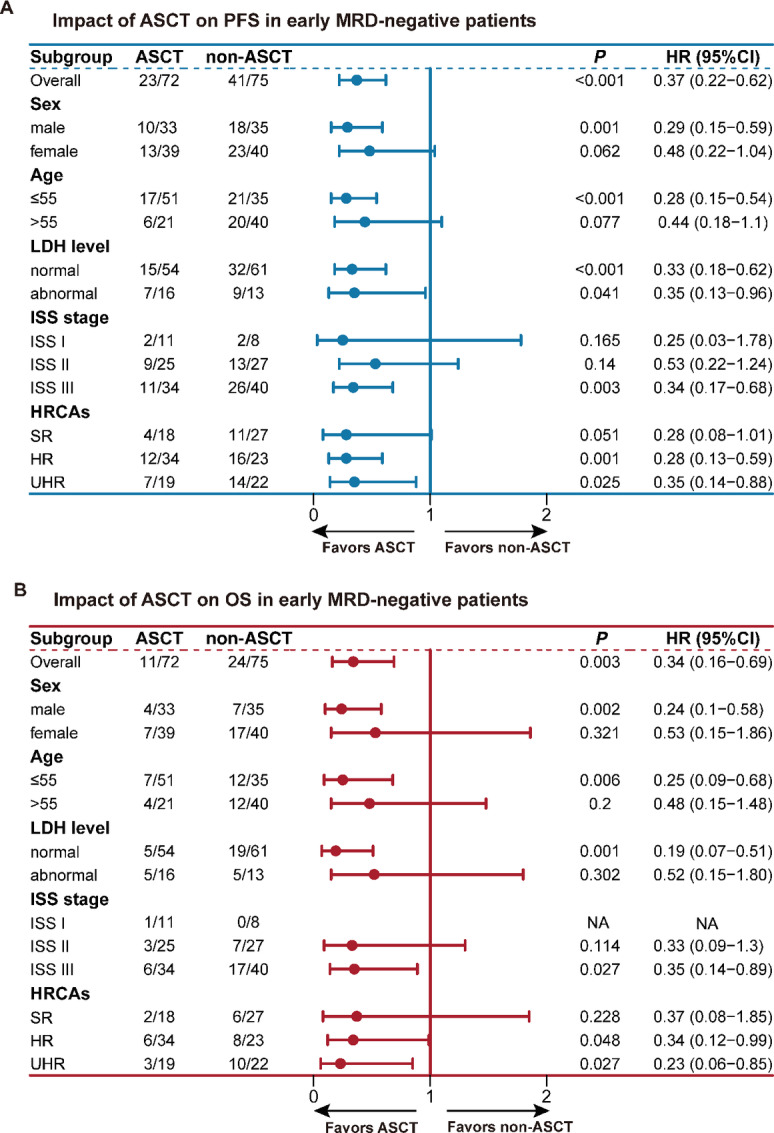
Subgroup analyses for impact of ASCT among early MRD-negative patients on PFS (**A**), OS (**B**). Abnormal LDH level: >247 U/L; ASCT: autologous stem cell transplant; early MRD-negative: achieving MRD-negative after induction treatment; HR: high-risk; HRCAs: high-risk cytogenetic abnormalities, including Del(17p), t(4;14), t(14;16), or Gain 1q; ISS: International Staging System; OS: overall survival; PFS: progression-free survival; SR: standard risk; UHR: ultra-high-risk.

When restricted to the early MRD-negative patients, the impact of ASCT was estimated in subgroup analyses in terms of sex, age, LDH level, ISS stage, and molecular risk. The subgroup analyses revealed that ASCT was predictive of an improved PFS in patients with aggressive features (ISS stage III and HRCAs; *P* < 0.05; Fig. 2A). In terms of OS, ASCT retained a favorable improvement in patients with ISS stage III, high-risk, and UHR cohorts (*P* < 0.05; Fig. 2B). For those patients that presented with early MRD-positive status, the subgroup analyses of PFS and OS were very similar in that ASCT could effectively improve prognosis in high-risk/ultra-high-risk cohorts apart from in those patients without HRCA (Supplementary Fig. S4). These data further emphasized the indispensable role of ASCT and the achievement of undetectable MRD in patients with HRCAs.

### Extension of MRD-Negative Duration by ASCT

**FIGURE 3:**
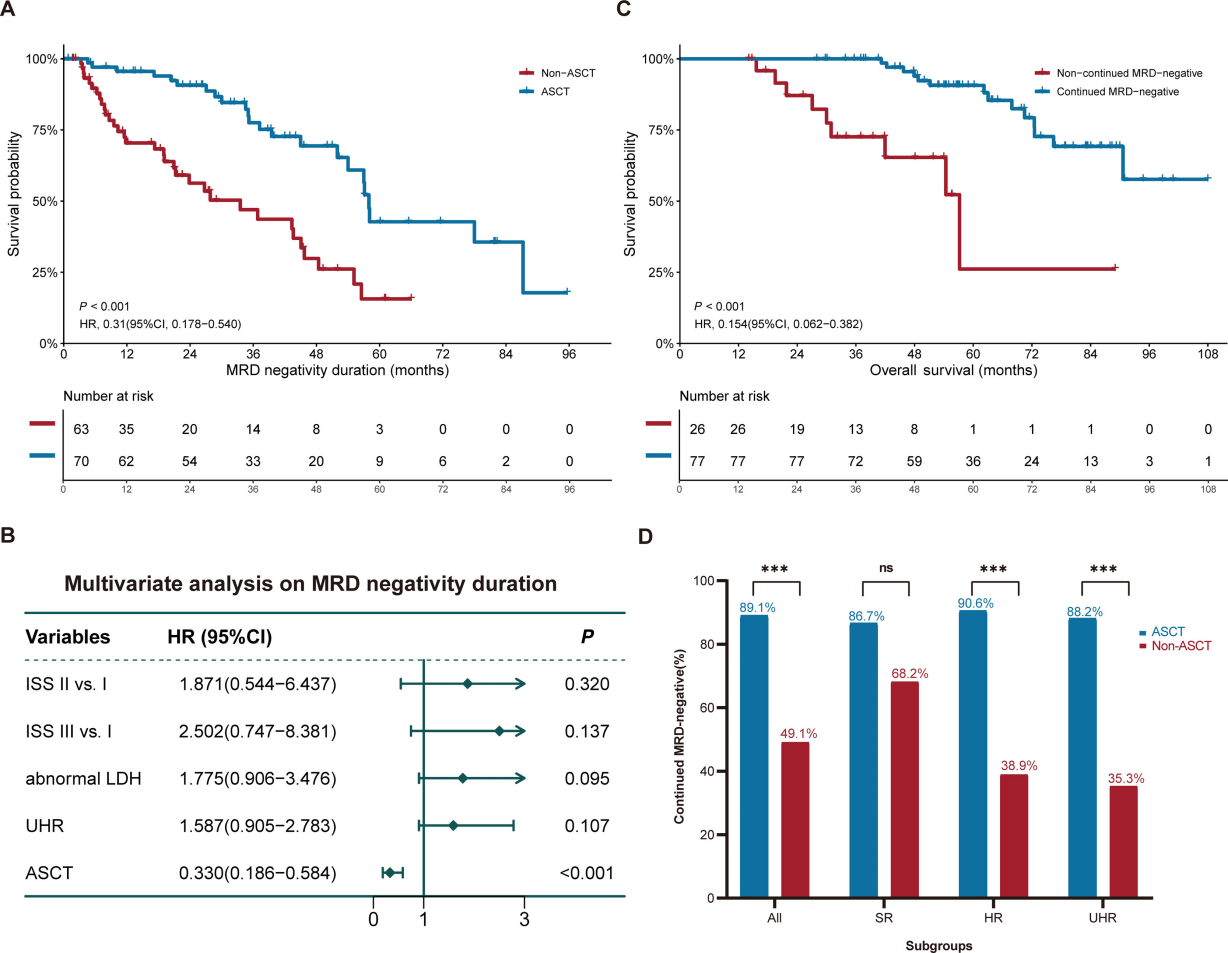
The impact of ASCT on MRD status duration among early MRD-negative patients: impact of ASCT on MRD-negative status duration (**A**); multivariate analysis for MRD-negative duration (**B**); impact of continued MRD-negative status (≥2 years) on OS (**C**); the impact of ASCT on the proportion of continued MRD-negative status (≥2 years) by different molecular risk groups (**D**). All: early MRD-negative cohort; ASCT: autologous stem-cell transplant; HR: high-risk cohort; OS: overall survival; SR: standard risk cohort; UHR: ultra-high-risk cohort.

In addition to its role in prognosis, we further investigated the correlation between ASCT and MRD status duration. Among patients who achieved early MRD-negative status, the ASCT cohort experienced an MRD-negative duration of 58.0 months (95% CI: 56.5–59.4 months), considerably longer than in those patients who did not receive ASCT (33.5 months, 95% CI: 17.9–49.2 months; *P* < 0.001; Fig. 3A). The predictors of MRD-negative duration were also analyzed by univariate and multivariate analyses. The results of this analysis revealed that ASCT (HR = 0.330; 95% CI, 0.186–0.584; *P* < 0.001) was the only independent favorable factor influencing MRD-negative duration (Fig. 3B; Supplementary Table S4).

Consistent with previous literature (18, 19), we observed that continued MRD-negative status for more than 2 years was strongly correlated with favorable prognosis among early MRD-negative patients (*P* < 0.001; Fig. 3C; Supplementary Fig. S5). As we expected, a total of 89.1% (57/64) patients in the ASCT cohort experienced continued MRD-negative duration (≥2 years), while the proportion was just 49.1% (28/57) in the no-ASCT group (*P* < 0.001). Subgroup analyses of diverse molecular risk further revealed that ASCT could effectively increase the proportion of continued MRD-negative status in patients within the high-risk (90.6% vs. 38.9%; *P* < 0.001) or UHR groups (88.2% vs. 35.3%; *P* < 0.001), but not in the SR group (86.7% vs. 68.2%; *P* = 0.198; Fig. 3D). It could therefore be concluded that the significantly improved survival outcomes in the ASCT cohort can be attributed to the extension of MRD-negative duration by ASCT, and that the benefit of ASCT was more crucial in patients with high-risk features.

### Prognostic Significance of Longitudinal MRD Status Before and After ASCT

**FIGURE 4:**
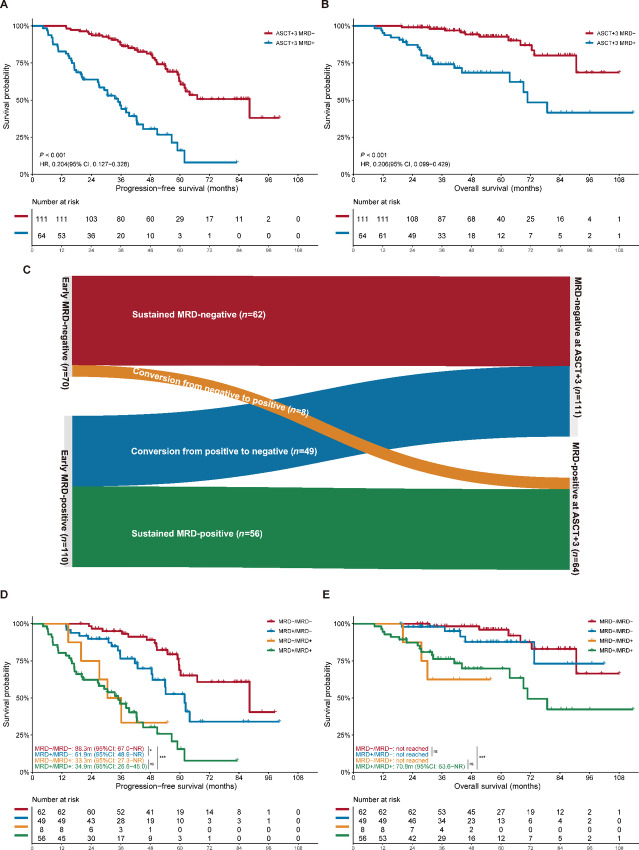
Impact of longitudinal MRD status before and after ASCT on prognosis: impact of MRD status at ASCT+3 on PFS (**A**), the impact of MRD status at ASCT+3 on OS (**B**); the distribution of different MRD status before and after ASCT (**C**); impact of longitudinal MRD status conversion on PFS (**D**); impact of longitudinal MRD status conversion on OS (**E**). ASCT: autologous stem-cell transplant; NR: not reached; NS: not significant; OS: overall survival; PFS: progression-free survival; *, *P* < 0.05; **, *P* < 0.01; ***, *P* < 0.001.

In addition to MRD status after induction treatment, we also interrogated the subsequent MRD status and its conversion after ASCT. Among patients in the ASCT cohort, MRD status 3 months after ASCT was available for 175 patients and a total of 111 (63.4%) patients achieved undetectable MRD. MRD-negative status at ASCT+3 was closely associated with a remarkable increase in the median PFS, from 34.9 months with detectable MRD to 88.3 months (HR: 0.204; 95% CI, 0.127–0.328; *P* < 0.001; Fig. 4A). Similarly, MRD-negative status at ASCT+3 was also predictive of improved OS in patients who received ASCT (not reached vs. 70.8 months; 0.206; 0.099–0.429; *P* < 0.001; Fig. 4B).

In terms of MRD status at different timepoints, diverse MRD status conversion patterns before and after ASCT were identified and investigated (Fig. 4C). Sixty-two (35.4%) patients were MRD-negative both pre-ASCT and at ASCT+3 timepoints. This sustained MRD-negative cohort had the most favorable survival rates, with a median PFS of 88.3 months while a median OS could not be reached. Meanwhile, the outcomes were the least favorable in the 56 (32.0%) patients who remained MRD-positive at both timepoints (PFS: 34.9 months; OS: 70.8 months; *P* < 0.001; Fig. 4D and E). However, the comparisons of baseline features between sustained MRD-negative and MRD-positive patients did not reveal obvious discrepancies as we expected and furthermore, the 17p deletion was more prevalent in the sustained MRD-negative cohort (21.3% vs. 5.4%; *P* = 0.015; Supplementary Table S5). Patients who converted from MRD-positive pre-ASCT to MRD-negative at ASCT+3 experienced a remarkably increased PFS compared with those patients MRD-positive at ASCT+3 (61.9 vs. 34.9 months; *P* < 0.001; Fig. 4D). Likewise, conversion from MRD-positive to MRD-negative status was additionally associated with similar improved OS as patients in the sustained MRD-negative cohort (*P* = 0.372; Fig. 4E), although the data may be constrained by limited follow-up times. Surprisingly, 8 (4.6%) patients who were MRD-negative before ASCT were observed to have detectable MRD at ASCT+3, and this MRD conversion pattern from negative to positive was predictive of the deterioration of PFS and OS (*P* < 0.001).

## Discussion

In this retrospective study, we observed that ASCT is significantly correlated with improved clinical outcomes independent of early MRD negativity, particularly in myeloma patients exhibiting aggressive features (high molecular risk or high tumor burden). This observation warrants validation through prospective clinical trials.

Multiple recent studies have proposed that undetectable MRD is the most important predictor for good prognosis in any subgroup of patients with multiple myeloma (1–3). Consistent with the literature, our results also recapitulate that early MRD status after treatment was strongly associated with an improvement in survival, which provided some evidence that applying intensified induction treatment (daratumumab + VRD/VTD) in patients with multiple myeloma would enhance early remission rate (10, 11). Interestingly, in our cohort, early MRD-negative status was more frequent in patients with HRCAs or those classified as R-ISS stage III. However, durable remission was difficult to attain in such patients with aggressive disease (20, 21). This phenomenon could be interpreted partially because aggressive myeloma cells are easier to reduce in number but not completely removed by treatment.

With the advancement of novel drugs in recent decades, the treatment pattern and response kinetics of myeloma has changed dramatically. In the GRIFFIN/CASSIOPEIA clinical trials, early MRD-negative status rates in the daratumumab cohort were considerably higher than the control group who received classical triplet combined regimens (VRD/VTD; refs. 10, 11). As achieving undetectable MRD has been reckoned as a surrogate endpoint or “curative status” in multiple myeloma, it is worth understanding whether patients without MRD could still benefit from ASCT or conversely be harmed by the side effects of high doses of melphalan.

In our study, we concluded that the prognoses of patients who received ASCT were significantly improved compared with those in the non-ASCT group among those with early MRD-negative status. Of note, the favorable prognostic effects of ASCT were more remarkable in patients with aggressive features (ISS stage III or HRCAs). These results reemphasized the favorable impact of ASCT even in the era of novel agents, particularly in high-risk patients with unsatisfactory survival outcomes. Nevertheless, the observed simultaneous improvements in PFS and OS associated with ASCT in our study are not consistent with the findings of two large prospective clinical trials (IFM2009/DETERMINATION). These trials demonstrated a significant extension in PFS but no substantial enhancement in OS among patients undergoing ASCT (6, 22). The observed discrepancy can be partially explained by variations in the proportion of high-risk individuals among the patient cohorts, specifically ISS stage III (this study: 48.6% vs. IFM2009: 18.0% vs. DETERMINATION: 13.3%). Furthermore, it is important to note that a substantial number of patients in the VRD-alone group of the IFM2009/DETERMINATION trial underwent second-line ASCT, deriving similar OS benefits compared with ASCT group. Therefore, additional studies are required in the future to comprehensively investigate and address the clinical question regarding the potential improvement on OS of ASCT in multiple myeloma.

The internal cause of prognosis improvement associated with transplant could be explained by patients exhibiting a more durable MRD-negative status in the ASCT cohort. Moreover, ASCT could potentially enhance remission depth through the killing of residual tumor cells by high-dose chemotherapy (9). Conversely, ASCT may prolong the MRD-negative duration by rebuilding the bone marrow immune microenvironment (23, 24). Several studies have further proposed that the persistence of undetectable MRD is correlated with normal immune microenvironment (25, 26). However, a subtle decline in MRD level (from 10^−6^ to 10^−8^ or lower) may not be observed by the limited detective sensitivity of flow cytometry. As such, new techniques such as next-generation sequencing and mass spectrometry should be applied for detecting MRD and describing minimal changes in MRD levels in the future (27, 28).

Furthermore, it is significant to carefully consider the toxicities of high-dose melphalan, which may increase the mutational burden at relapse (29) and cause the escalation of therapeutic-related malignancies. Recent investigations have shed light on the underlying mechanisms, and have revealed that high-dose melphalan has the capacity to induce mutational signatures associated with the development of second malignancies (30–32). In addition, ASCT can potentially lead to reinfusion of clonal hematopoiesis (CH) clones and melphalan could induce immunosuppression which facilitates the second tumors (33). Hence, it may be necessary to consider performing genetic screening for CH for patients with transplant-eligible multiple myeloma. This screening could comprehensively evaluate the survival benefits and potential toxicity associated with high-dose melphalan, thereby enabling an optimal treatment regimen.

The duration of MRD-negative status was positively correlated with prognosis in patients with multiple myeloma, a finding that has been validated in Pollux/Castle and Maia/Alcyone studies (18, 19). Similarly, in our study, such patients with an MRD-negative status duration of ≥2 years experienced significantly longer lifetimes than those with less than 2 years of continued MRD-negativity. This finding further suggests that dynamic MRD status is more critical than static monitoring (34).

Importantly, the enhancement of MRD duration by ASCT was more evident in high-risk/ultra-high-risk cohorts, exhibiting that those patients who received ASCT had a significantly higher proportion of MRD-negative status duration ≥2 years than those in the non-ASCT group. However, this phenomenon was not observed in patients without HRCAs. In the FORTE study, similar conclusions were obtained in that ASCT could decrease the frequency of MRD reappearance in the R-ISS II/III cohort but not R-ISS I patients (35). Collectively, ASCT exerts vital effects that result in improving the prognosis of patients with high-risk multiple myeloma, which may be associated with the fact that high-risk patients often exhibit rapid remission during induction treatment followed by early progression (20, 36). Deep remission acquired early is difficult to sustain, while intensive consolidation treatment, such as ASCT, was observed to improve the duration of response.

In our study, the prognostic impact of MRD status at ASCT+3 is consistent with the results from other recent studies (1, 3, 12, 37). In the Myeloma XI clinical trial, MRD-negative status at ASCT +3 was correlated with a lower risk of progression and death (HRs: 0.47 and 0.59, respectively; ref. 12). It was also noted that the diverse patterns of MRD conversion before and after ASCT is of great clinical significance. In patients with MRD-positive status after induction treatment, MRD disappearance after ASCT was associated with good survival outcomes that were comparable with sustained MRD-negative patients. Moreover, re-emergence of MRD after ASCT predicted an unfavorable prognosis in early MRD-negative patients. These results emphasize the value of continued MRD monitoring among those treated with ASCT when estimating the efficacy of sequential treatment in prospective trials. Furthermore, it is feasible that therapeutic options for multiple myeloma could be stratified by the longitudinal MRD status (12). When MRD reemerges after ASCT, intensified consolidation or maintenance therapy should be applied to control disease. As such, further prospective clinical trials should be designed to estimate the value of dynamic MRD monitoring on therapeutic options.

This study should be interpreted considering its strengths and limitations. One limitation of our study is that the median MRD detection sensitivity was 4 × 10^−5^ and MRD status did not include peripheral blood or imaging. Considering the focal distribution of multiple myeloma lesions, simple bone marrow MRD status may not be entirely comprehensive for the overall evaluation of remission. In addition, the validity of results may be affected partially by our retrospective design apart from in uniform therapy as clinical trials. However, the heterogeneity of induction treatment identified herein could also support the generalizability of our findings. Finally, the induction treatment patterns in our cohort did not include the more effective four-drug combination therapy. Therefore, it is necessary in the future to analyze a larger sample size and design prospective clinical trials to further demonstrate the relevant conclusions from this study.

In summary, our findings demonstrated the improved clinical importance of ASCT in patients with transplant-eligible myeloma, irrespective of early MRD status after induction treatment, with a particular emphasis on high-risk patients. These findings call for rigorous validation via prospective clinical trials.

## Supplementary Material

Figure S1 The workflow of patient selection in this study.

Figure S2 The prognostic impact of ASCT among all patients

Figure S3 The prognostic impact of ASCT and early MRD status by molecular risk groups

Figure S4 Subgroup analyses for prognostic impact of ASCT

Figure S5 The impact of continued MRD-negative status (≥2 years) on PFS

Table S1 Patient Characteristics, Treatments, and MRD detective methods: ASCT vs. Non-ASCT

Table S2 The impact of ASCT on PFS and OS among all patients

Table S3 Univariate analyses of PFS and OS in 407 transplant-eligible myeloma patients

Table S4 Univariate and multivariate analyses of MRD negativity duration

Table S5 Patient and Treatment Characteristics in ASCT cohort: Sustained MRD-negative vs. Sustained MRD-positive
