# Supplementary figures and images for: Clinical Benefit of Autologous Stem Cell Transplantation for Patients with Multiple Myeloma Achieving Undetectable Minimal Residual Disease after Induction Treatment
Source: Cancer Res Commun. 2023 Sep 6;3(9):1770–80. doi: 10.1158/2767-9764.CRC-23-0185 (PMC10481879; doi:10.1158/2767-9764.CRC-23-0185)

Figure S1

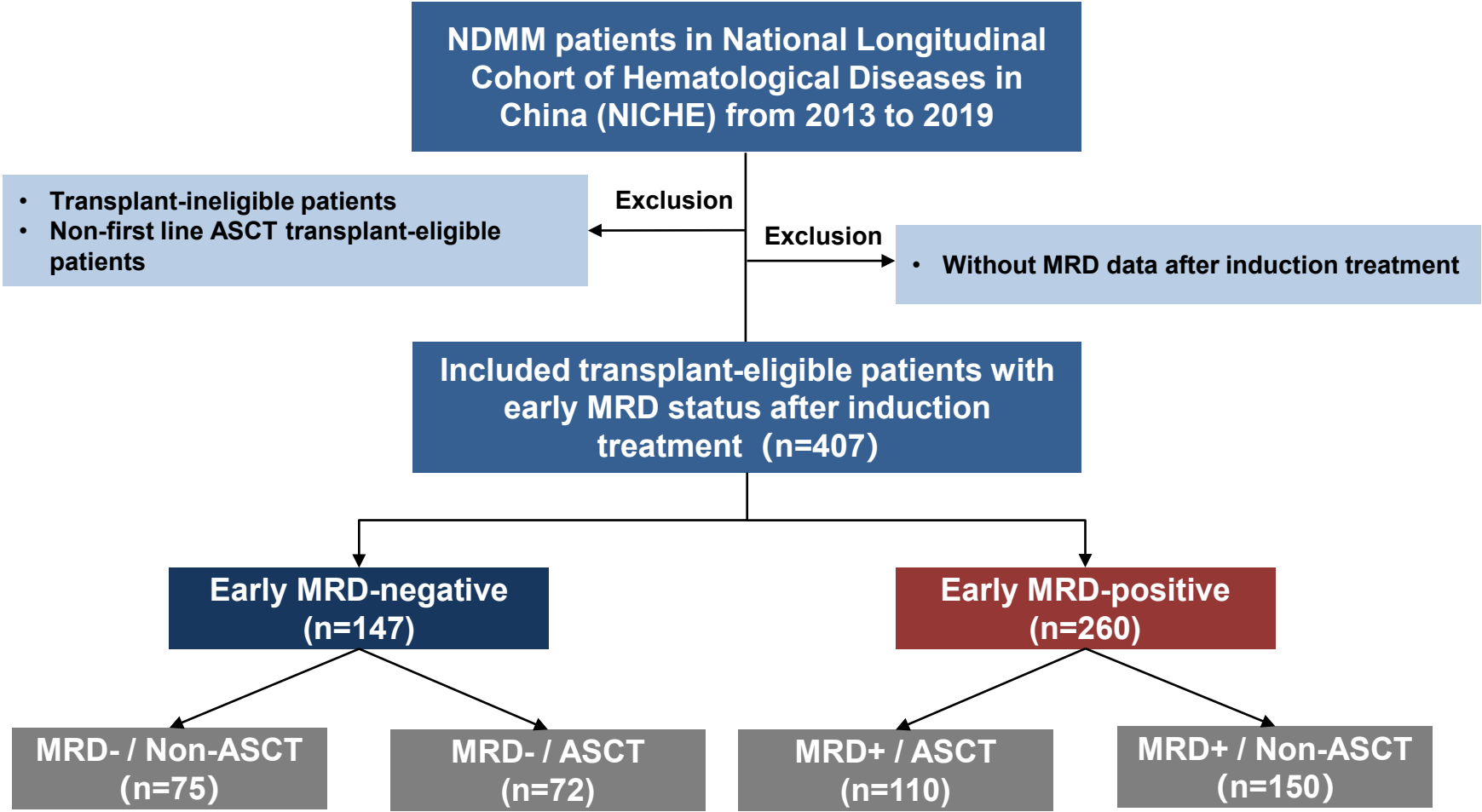

Figure S1: The workflow of patient selection in this study.

Supplement: Figure S1 — The workflow of patient selection in this study. [file crc-23-0185-s01.pdf]
