# Supplementary material for: Clinical Benefit of Autologous Stem Cell Transplantation for Patients with Multiple Myeloma Achieving Undetectable Minimal Residual Disease after Induction Treatment
Source: Cancer Res Commun. 2023 Sep 6;3(9):1770–80. doi: 10.1158/2767-9764.CRC-23-0185 (PMC10481879; doi:10.1158/2767-9764.CRC-23-0185)
Supplement: Figure S2 — The prognostic impact of ASCT among all patients [file crc-23-0185-s02.pdf]

Figure S2

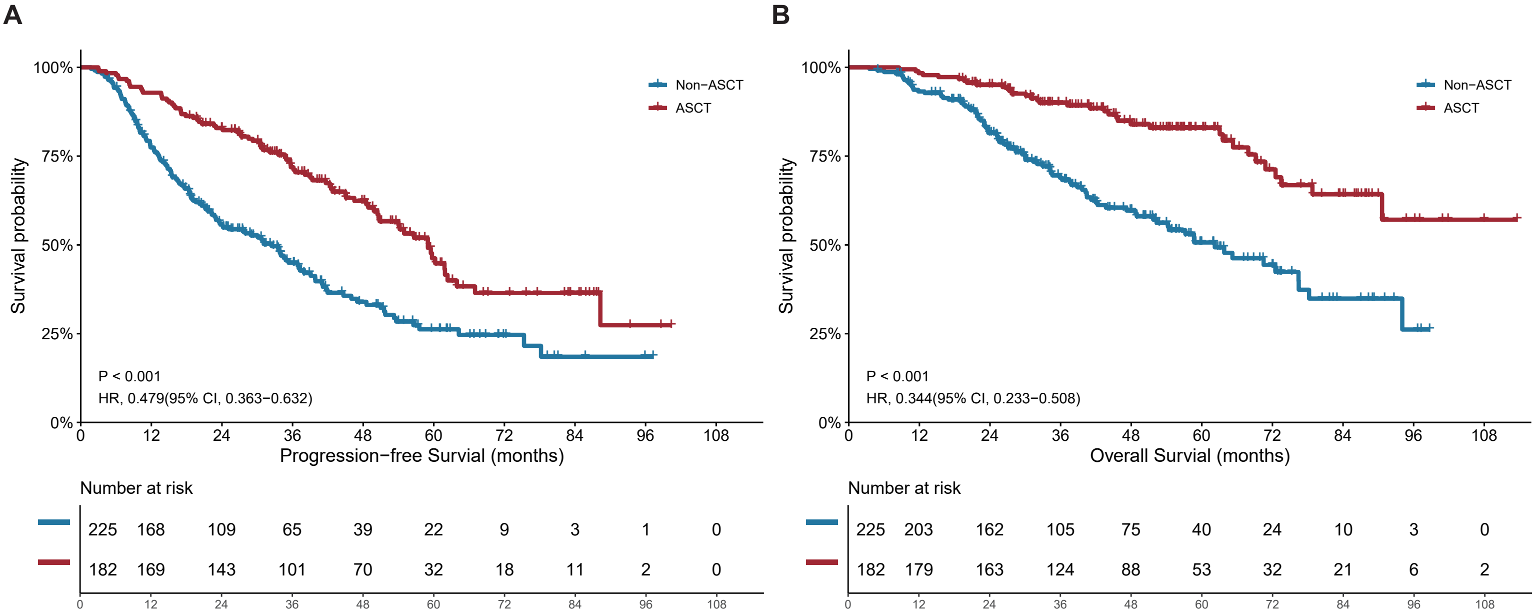

Figure S2: The prognostic impact of ASCT among all patients: (A) impact of ASCT on PFS, (B) impact of ASCT on OS.
ASCT: autologous stem-cell transplant; PFS: progression-free survival; OS: overall survival.
