# Supplementary material for: Clinical Benefit of Autologous Stem Cell Transplantation for Patients with Multiple Myeloma Achieving Undetectable Minimal Residual Disease after Induction Treatment
Source: Cancer Res Commun. 2023 Sep 6;3(9):1770–80. doi: 10.1158/2767-9764.CRC-23-0185 (PMC10481879; doi:10.1158/2767-9764.CRC-23-0185)
Supplement: Figure S3 — The prognostic impact of ASCT and early MRD status by molecular risk groups [file crc-23-0185-s03.pdf]

Figure S3

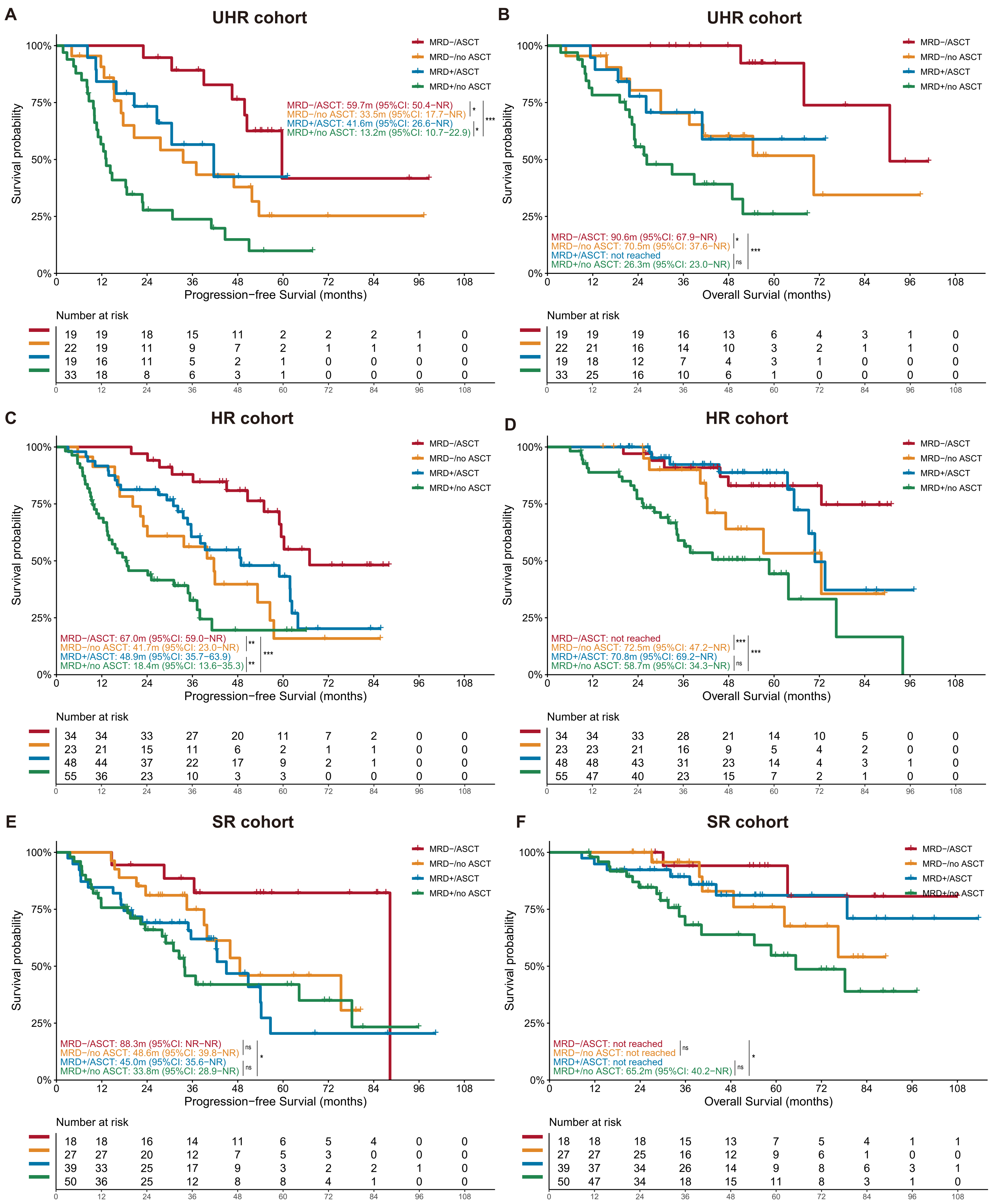

Figure S3: The prognostic impact of ASCT and early MRD status by molecular risk groups: (A) impact on PFS in HR group; (B) impact on OS in HR group; (C) impact on PFS in UHR group; (D) impact on OS in UHR group; (E) impact on PFS in SR group; (F) impact on OS in SR group.
ASCT: autologous stem-cell transplant; early MRD status: MRD status after induction treatment; PFS: progression-free survival; OS: overall survival; HR: high-risk; UHR: ultra-high-risk; SR: standard risk. NR: not reached; NS: not significant; *: P<0.05; **: P<0.01; ***: P<0.001.
