# Supplementary material for: Clinical Benefit of Autologous Stem Cell Transplantation for Patients with Multiple Myeloma Achieving Undetectable Minimal Residual Disease after Induction Treatment
Source: Cancer Res Commun. 2023 Sep 6;3(9):1770–80. doi: 10.1158/2767-9764.CRC-23-0185 (PMC10481879; doi:10.1158/2767-9764.CRC-23-0185)
Supplement: Figure S4 — Subgroup analyses for prognostic impact of ASCT [file crc-23-0185-s04.pdf]

Figure S4

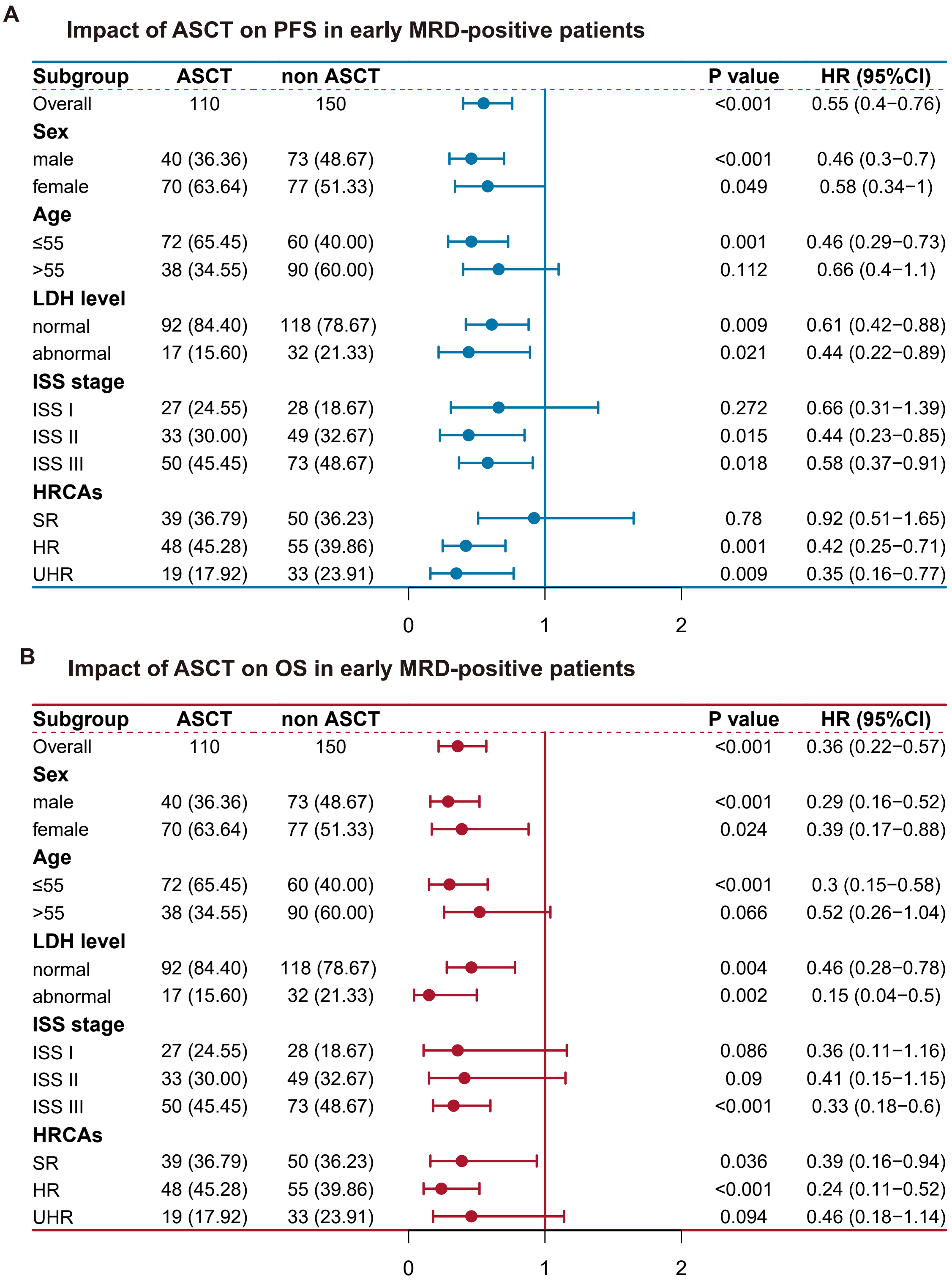

Figure S4: Subgroup analyses for prognostic impact of ASCT: (A) impact of ASCT on PFS among early MRD-positive patients, (B) impact of ASCT on OS among early MRD-positive patients.
