# Supplementary material for: Clinical Benefit of Autologous Stem Cell Transplantation for Patients with Multiple Myeloma Achieving Undetectable Minimal Residual Disease after Induction Treatment
Source: Cancer Res Commun. 2023 Sep 6;3(9):1770–80. doi: 10.1158/2767-9764.CRC-23-0185 (PMC10481879; doi:10.1158/2767-9764.CRC-23-0185)
Supplement: Figure S5 — The impact of continued MRD-negative status (≥2 years) on PFS [file crc-23-0185-s05.pdf]

Figure S5

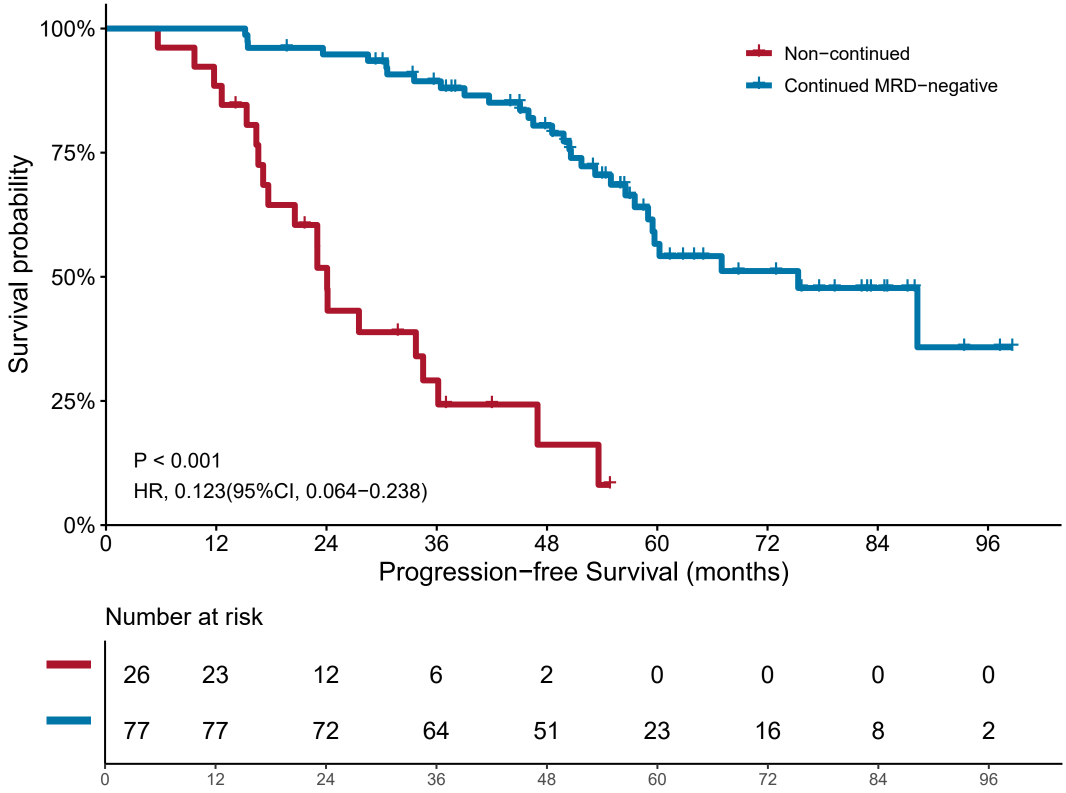

Figure S5: The impact of continued MRD-negative status (≥2 years) on PFS; PFS: progression-free survival.
