# Supplementary material for: Clinical Benefit of Autologous Stem Cell Transplantation for Patients with Multiple Myeloma Achieving Undetectable Minimal Residual Disease after Induction Treatment
Source: Cancer Res Commun. 2023 Sep 6;3(9):1770–80. doi: 10.1158/2767-9764.CRC-23-0185 (PMC10481879; doi:10.1158/2767-9764.CRC-23-0185)
Supplement: Table S1 — Patient Characteristics, Treatments, and MRD detective methods: ASCT vs. Non-ASCT [file crc-23-0185-s06.pdf]

Table S1. Patient Characteristics, Treatments, and MRD detective methods: ASCT vs. Non-ASCT

Characteristics (%)	All cohort (n=407)	ASCT (n=182)	Non-ASCT (n=225)	*P value*
**Age (median; years)**	55(22-65)	52(31-64)	57(22-65)	<0.001
**Sex**	M:226(55.5), F:181(44.5)	M:109(59.9), F:73(40.1)	M:117(52.0), F:108(48.0)	0.132
**M-component**				0.824
**IgG**	194(47.7)	83 (45.6)	111 (49.3)	
**IgA**	87(21.4)	39 (21.4)	48 (21.3)	
**Light chain**	88(21.6)	43 (23.6)	45 (20.0)	
**Others**	38(9.3)	17 (9.4)	21 (9.3)	
**ISS staging**				0.414
**I**	74(18.3)	38 (21.1)	36 (16.0)	
**II**	134(33.1)	58 (32.2)	76 (33.8)	
**III**	197(48.6)	84 (46.7)	113 (50.2)	
**Missing**	2	2	0	
**RISS staging**				0.095
**I**	53(13.1)	30 (16.7)	23(10.3)	
**II**	244(60.4)	102 (56.7)	142(63.4)	
**III**	107(26.5)	48 (26.7)	59(26.3)	
**Missing**	3	2	1	
**Cytogenetic abnormality**				
**Del(17p)**	56/402(13.9)	20/181 (11.0)	36/221(16.3)	0.149
**t(4;14)**	77/387(19.9)	39/177 (22.0)	38/210(18.1)	0.372
**t(14;16)**	17/386(4.4)	8/177 (4.5)	9/209(4.3)	1.000
**Gain 1q**	215/405(53.1)	98/182 (53.8)	117/223(52.5)	0.841
**HRCAs**				0.182
**0**	134(34.6)	57(32.2)	77(36.7)	
**1**	160(41.4)	82(46.3)	78(37.1)	
**≥2**	93(24.0)	38(21.5)	55(26.2)	
**Missing**	20	5	15	
**Induction treatment**				0.063
**PIs based**	299(73.5)	128(70.3)	171(76.0)	
**IMiDs based**	7(1.7)	1(0.5)	6(2.7)	
**PIs+IMiDs based**	101(24.8)	53(29.1)	48(21.3)	
**Early undetectable MRD**	147(36.1)	72(39.6)	75(33.3)	0.213
**MRD detective method**				0.109
**MFC**	222(54.5)	91(50.0)	131(58.2)	
**EuroFlow**	185(45.4)	91(50.0)	94(41.8)	

Abbreviations: ISS: International Staging System; R-ISS: Revised International Staging System; HRCAs: high risk cytogenetic abnormalities, including Del(17p), t (4;14), t (14;16), or Gain 1q; PIs: proteasome inhibitors; IMiDs: immunomodulators; MFC: multiparametric flow cytometry.
