# Supplementary material for: Clinical Benefit of Autologous Stem Cell Transplantation for Patients with Multiple Myeloma Achieving Undetectable Minimal Residual Disease after Induction Treatment
Source: Cancer Res Commun. 2023 Sep 6;3(9):1770–80. doi: 10.1158/2767-9764.CRC-23-0185 (PMC10481879; doi:10.1158/2767-9764.CRC-23-0185)
Supplement: Table S2 — The impact of ASCT on PFS and OS among all patients [file crc-23-0185-s07.pdf]

Table S2 The impact of ASCT on PFS and OS among all patients

Subgroup	PFS (months)	OS (months)
**ASCT group**	59.0 (95%CI: 50.6-63.9)	Not reached
**Non-ASCT group**	32.5 (95%CI: 23.6-38.0)	62.2 (95%CI: 51.7-76.5)

Abbreviations: ASCT: autologous stem-cell transplant; PFS: progression-free survival
