# Supplementary material for: Clinical Benefit of Autologous Stem Cell Transplantation for Patients with Multiple Myeloma Achieving Undetectable Minimal Residual Disease after Induction Treatment
Source: Cancer Res Commun. 2023 Sep 6;3(9):1770–80. doi: 10.1158/2767-9764.CRC-23-0185 (PMC10481879; doi:10.1158/2767-9764.CRC-23-0185)
Supplement: Table S3 — Univariate analyses of PFS and OS in 407 transplant-eligible myeloma patients [file crc-23-0185-s08.pdf]

Table S3 Univariate analyses of PFS and OS in 407 transplant-eligible myeloma patients

**Factors**	**PFS**		**OS**	
	**HR (95% CI)**	***P value***	**HR (95% CI)**	***P value***
**ISS stage at diagnosis**				
**II vs. I**	1.171(0.771-1.783)	0.458	1.102(0.598-2.030)	0.755
**III vs. I**	1.739(1.181-2.559)	0.005	2.525(1.474-4.326)	<0.001
**Serum LDH level at diagnosis**				
**abnormal vs. normal**	1.902(1.399-2.584)	<0.001	2.218(1.495-3.291)	<0.001
**Cytogenetic abnormality at diagnosis**				
**HR vs. SR**	1.265(0.914-1.751)	0.156	1.380(0.884-2.154)	0.156
**UHR vs. SR**	1.561(1.085-2.246)	0.016	2.260(1.412-3.617)	<0.001
**PIs+IMiDs based induction treatment**				
**yes vs. no**	0.756(0.527-1.083)	0.127	1.107(0.693-1.768)	0.671
**MRD status after induction treatment**				
**negative vs. positive**	0.447(0.333-0.600)	<0.001	0.473(0.320-0.700)	<0.001
**ASCT status**				
**yes vs. no**	0.479(0.363-0.632)	<0.001	0.344(0.233-0.508)	<0.001

The indexes with P < 0.1 from univariate analysis were included in the multivariate analysis. Abbreviations: HR=hazard ratio; ISS: International staging system; Abnormal LDH: lactate dehydrogenase>247 U/L; HRCAs: high risk cytogenetic abnormalities, including Del(17p), t (4;14), t (14;16), or Gain 1q; HR: high risk, with one HRCAs; SR: standard risk, without any HRCAs; UHR: ultra-high risk, with more than one HRCAs; PIs: proteasome inhibitors; IMiDs: immunomodulators; ASCT: autologous stem cell transplantation.
