# Supplementary material for: Clinical Benefit of Autologous Stem Cell Transplantation for Patients with Multiple Myeloma Achieving Undetectable Minimal Residual Disease after Induction Treatment
Source: Cancer Res Commun. 2023 Sep 6;3(9):1770–80. doi: 10.1158/2767-9764.CRC-23-0185 (PMC10481879; doi:10.1158/2767-9764.CRC-23-0185)
Supplement: Table S4 — Univariate and multivariate analyses of MRD negativity duration [file crc-23-0185-s09.pdf]

Table S4. Univariate and multivariate analyses of MRD negativity duration

Variables	Univariate Analysis		Multivariate Analysis	
	HR (95%CI)	P Value	HR (95%CI)	P Value
**ISS stage**				
**ISS II vs. I**	3.055(0.910-10.260)	0.071	1.871(0.544-6.437)	0.320
**ISS III vs. I**	3.942(1.199-12.960)	0.024*	2.502(0.747-8.381)	0.137
**Abnormal LDH**	1.760(0.908-3.412)	0.094	1.775(0.906-3.476)	0.095
**HRCAs**				
**HR vs. SR**	1.329(0.659-2.683)	0.427		
**UHR vs. SR**	1.993(0.979-4.06)	0.057	1.587(0.905-2.783)	0.107
**PIs+IMiDs-based**	0.568(0.268-1.20)	0.140		
**ASCT**	0.310(0.178-0.540)	<0.001*	0.330(0.186-0.584)	<0.001*

The indexes with P < 0.1 from univariate analysis were included in the multivariate analysis.
Abbreviations: HR=hazard ratio; ISS: International staging system; Abnormal LDH: lactate dehydrogenase>247 U/L; HRCAs: high risk cytogenetic abnormalities, including Del(17p), t (4;14), t (14;16), or Gain 1q; HR: high risk; SR: standard risk; UHR: ultra-high risk; PIs: proteasome inhibitors; IMiDs: immunomodulators; ASCT: autologous hematopoietic stem cell transplantation.
*Statistically significant
