# Supplementary material for: Clinical Benefit of Autologous Stem Cell Transplantation for Patients with Multiple Myeloma Achieving Undetectable Minimal Residual Disease after Induction Treatment
Source: Cancer Res Commun. 2023 Sep 6;3(9):1770–80. doi: 10.1158/2767-9764.CRC-23-0185 (PMC10481879; doi:10.1158/2767-9764.CRC-23-0185)
Supplement: Table S5 — Patient and Treatment Characteristics in ASCT cohort: Sustained MRD-negative vs. Sustained MRD-positive [file crc-23-0185-s10.pdf]

Table S5. Patient and Treatment Characteristics in ASCT cohort: Sustained MRD-negative vs. Sustained MRD-positive

Characteristics (%)	Sustained MRD-negative (n=62)	Sustained MRD-positive (n=56)	P value
**Age (median; years)**	52(31-64)	52(36-64)	0.897
**Sex**	M:33, F:29	M:38, F:18	0.133
**M-component**			0.487
**IgG**	26 (41.9)	23 (41.1)	
**IgA**	18 (29.0)	13 (23.2)	
**Light chain**	15 (24.2)	13 (23.2)	
**Others**	3 (4.8)	7 (12.5)	
**ISS staging**			0.786
**I**	11 (18.0)	13 (23.2)	
**II**	21 (34.4)	18 (32.1)	
**III**	29 (47.5)	25 (44.6)	
**Missing**	1	0	
**RISS staging**			0.279
**I**	8 (13.1)	11 (19.6)	
**II**	35 (57.4)	35 (62.5)	
**III**	18 (29.5)	10 (17.9)	
**Missing**	1	0	
**Cytogenetic abnormality**			
**Del(17p)**	13/61(21.3)	3/56(5.4)	0.015
**t(4;14)**	15/61(24.6)	7/56(12.5)	0.104
**t(14;16)**	4/61(6.6)	4/56(7.1)	1.000
**Gain 1q**	33/62(53.2)	29/56(51.8)	1.000
**HRCAs**			0.140
**0**	16(26.2)	24(42.9)	
**1**	28(45.9)	22(39.3)	
**≥2**	17(27.9)	10(17.9)	
**Missing**	1	0	
**Induction treatment**			0.542
**PIs based**	43(69.4)	42 (75.0)	
**IMiDs based**	0(0.0)	0(0.0)	
**PIs+IMiDs based**	19(30.6)	14 (25.0)	

Abbreviations: ISS: International Staging System; R-ISS: Revised International Staging System; HRCAs: high risk cytogenetic abnormalities, including Del(17p), t (4;14), t (14;16), or Gain 1q; PIs: proteasome inhibitors; IMiDs: immunomodulators.
